# Transcutaneous Electrical Nerve Stimulation for Muscle Recovery: Insights into Delayed Onset Muscle Soreness

**DOI:** 10.3390/clinpract15090157

**Published:** 2025-08-28

**Authors:** Sebastian Szajkowski, Jarosław Pasek, Grzegorz Cieślar

**Affiliations:** 1 Faculty of Medical Sciences, Warsaw Medical Academy of Applied Sciences, 8 Rydygiera St., 01-793 Warszawa, Poland; sebastianszajkowski@wp.pl; 2 Collegium Medicum im dr Władysława Biegańskiego, Jan Długosz University in Częstochowa, 13/15 Armii Krajowej St., 42-200 Częstochowa, Poland; 3 Department of Internal Medicine, Angiology and Physical Medicine, Faculty of Medical Sciences in Zabrze, Medical University of Silesia in Katowice, 15 Stefana Batorego St., 41-902 Bytom, Poland; cieslar1@o2.pl

**Keywords:** muscle soreness, TENS, electrical nerve stimulation, recovery, myotonometry

## Abstract

**Background:** Delayed onset muscle soreness (DOMS) frequently occurs after engaging in strenuous physical activity. The manifestation of DOMS is often associated with changes in the biomechanical and viscoelastic characteristics of the affected muscles. **Materials and Methods:** Forty participants were enrolled and randomly assigned to two groups: the intervention group receiving transcutaneous electrical nerve stimulation (TENS, *n* = 20) and a control group (*n* = 20). A fatigue-inducing protocol targeting the gastrocnemius muscle was implemented to elicit DOMS. The effectiveness of TENS was assessed by evaluating alterations in the biomechanical and viscoelastic properties of the muscle. Pain intensity was recorded using the Numeric Rating Scale (NRS) at five time points: before the study began, three times during the intervention, and once at the conclusion of the study. **Results:** No statistically significant changes have been found regarding muscle tone (*p* = 0.162) and stiffness (*p* = 0.212) in Group 1. However, a statistically significant lower level of stiffness in Group 1 after the end of therapy has been detected (*p* = 0.008). Decrement values decreased statistically significantly, both in Group 1 (*p* = 0.015) and in Group 2 (*p* = 0.014). There were no statistically significant differences in decrement level between Group 1 and 2. Relaxation and creep decreased statistically insignificantly in both groups. At the end of the observation period (Day 4), statistically significant (*p* = 0.027) lower pain intensity was observed in Group 1. **Conclusions:** It has been demonstrated that TENS has had limited effectiveness in restoring baseline biomechanical and viscoelastic parameters of muscles that undergo changes during DOMS. TENS significantly relieves pain symptoms occurring in DOMS.

## 1. Introduction

Delayed onset muscle soreness (DOMS) is commonly experienced following novel physical exercise, especially when the exercise involves repeated eccentric muscle contractions. The soreness typically lasts between 24 and 72 h and is associated with a limited range of motion and muscular weakness. The peak intensity of symptoms usually occurs 48 h after the exercise [1]. Strenuous physical activity typically causes temporary micro-damages in skeletal muscles. These are accompanied by intracellular swelling, the displacement of cell nuclei, and the fragmentation of the cytoskeleton, particularly of desmin and dystrophin. Following muscle-damaging exercise, an acute inflammatory response occurs. This response includes the sequential release of interleukin (IL) 1 beta, IL-6, tumor necrosis factor-alpha (TNF-alpha), neutrophils and macrophages, and elevated levels of creatine kinase (CK) and lactate dehydrogenase (LDH) in the blood [2]. All of this contributes to local swelling, pressure on pain receptors, and increased pain perception. Pain is also associated with the sensitization of nociceptors by inflammatory mediators, altered nerve conduction in the affected muscles, and the heightened sensitivity of nerve endings. The inflammatory condition clinically manifests as DOMS. DOMS is usually felt when the affected muscles are moved, stretched, or palpated, and is often accompanied by increased tenderness and stiffness [1].

There are numerous approaches to treat DOMS, including non-steroidal anti-inflammatory drugs (NSAIDs) [3] and physiotherapeutic methods [4]. Among the available therapies, transcutaneous electrical nerve stimulation (TENS) is also used. The devices employed deliver electrical impulses through electrodes placed on the skin surface near nerves or at trigger points. The therapy is meant to increase blood circulation, decrease muscular tone, and induce anesthesia by activating peripheral nerves. At the peripheral level, TENS modulates pain by selectively activating large-diameter, low-threshold A-β afferents or small-diameter, high-threshold A-δ fibers. This afferent stimulation attenuates nociceptive signaling, thereby contributing to analgesia [5,6]. Furthermore, TENS has been shown to elevate levels of β-endorphins and methionine-enkephalin, endogenous opioids that bind to opioid receptors. Activation of these receptors inhibits the release of excitatory neurotransmitters such as glutamate and substance P within the spinal cord, resulting in reduced neuronal excitability and a potential decrease in central sensitization risk [7]. Recent studies have found that the most effective recovery methods are those which improve blood circulation, fluid filtration, or reabsorption in microcirculation. These include active recovery (AR), AR with added whole-body electromyostimulation (WB-EMS), neuromuscular electrical stimulation (NMES), and foam rolling (FR) [8,9]. A considerable number of randomized clinical trials of electrical stimulation’s effect on DOMS have been published [10,11,12,13]. However, there is still no consensus on the potential effects of TENS for the prevention or treatment of DOMS.

Myotonometry is a non-invasive technique used to quantitatively assess the mechanical (tone, stiffness, and elasticity) and viscoelastic (relaxation and creep) properties of muscle and connective tissues, which are often altered in the context of delayed onset muscle soreness (DOMS). This method has been shown to effectively substitute for subjective palpation-based assessments and is well-documented in the scientific literature [14,15]. The MyotonPro device (Myoton Ltd., Tallinn, Estonia) has demonstrated high reliability in quantifying muscle stiffness, with intraclass correlation coefficients (ICCs) ranging from 0.898 to 0.986 [16,17].

### Aim of the Study

The present study aims to investigate the effect of TENS on biomechanical and viscoelastic properties of gastrocnemius muscle and pain intensity after concentric–eccentric exercises resulting in the development of DOMS.

## 2. Material and Methods

The study enrolled 40 healthy volunteers (18 men and 22 women) who met the following inclusion criteria: age between 20 and 50 years, identification as either male or female, and a body mass index (BMI) ranging from 18.5 to 29.9 kg/m^2^. All participants reported a low level of physical activity and were not engaged in regular sports training. The decision to include individuals within a narrow age and BMI range aimed to minimize the potential influence of age-related morphological and biomechanical changes in muscle tissue. Exclusion criteria comprised any injuries treated within the past three months, recent musculoskeletal trauma, skin injuries or undiagnosed dermatological conditions at measurement sites, self-reported fatigue, fever, the presence of chronic diseases, or current use of medications. Additionally, participants were instructed to abstain from any physical exertion for at least 72 h prior to and throughout the duration of the study. All participants were informed of their right to withdraw from the study at any time, without providing a reason.

### 2.1. Study Design

**Figure 1:**
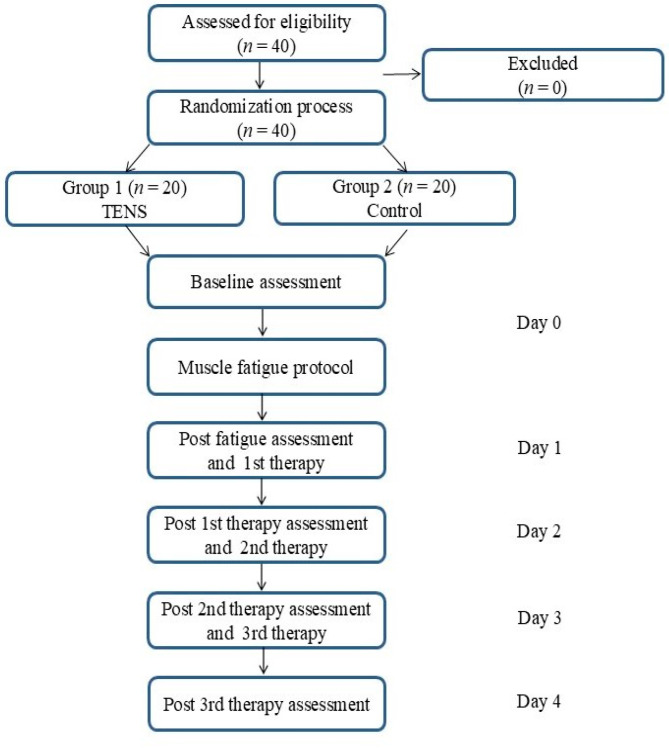
Study design.

This prospective clinical trial included forty healthy volunteers who were randomly assigned to one of two groups using simple 1:1 randomization generated via the Randomizer.org website. Group 1 received transcutaneous electrical nerve stimulation (TENS; *n* = 20; 9 men and 11 women), while Group 2 served as the control group (*n* = 20; 9 men and 11 women). The experimental protocol was structured as follows: Day 0—baseline assessment using a myotonometer and a pain intensity scale, followed by the administration of a muscle fatigue protocol; Day 1—post-fatigue assessment with the myotonometer and pain evaluation prior to the first therapeutic intervention, which was then performed; Days 2 and 3—myotonometric and pain assessments were conducted prior to therapeutic intervention, which followed the procedures applied on the previous day; Day 4—final measurements of muscle mechanical properties and pain intensity. In total, each participant underwent three therapeutic interventions (Days 1–3), five myotonometric assessments, and five pain intensity evaluations (Days 0–4) (see Figure 1). A 24 h recovery period was maintained between consecutive interventions and measurements to ensure adequate rest. All procedures were conducted between 09:00 and 14:00, with strict adherence to participant scheduling. Before inclusion in the study, all participants provided written informed consent. The testing environment was controlled and consistent throughout the study, with ambient temperature maintained at 22 °C and relative humidity at 50%.

### 2.2. Calf Raises—Muscle Fatigue Protocol

Participants were instructed to place the forefoot of their non-dominant limb at the edge of a step and perform single-leg heel raises, initiating with maximal plantar flexion of the ankle joint, followed by controlled lowering into maximal dorsiflexion, as previously described in the literature [18,19]. To standardize the movement cadence, a metronome set at 1.33 Hz was used, providing a cue for one quick plantar flexion phase followed by four slower eccentric lowering phases. To assist with balance, participants were permitted to lightly touch a wall positioned in front of them using their fingertips. Prior to the test, each participant was familiarized with the exercise protocol. The task was performed continuously until volitional exhaustion. Exhaustion was defined as the occurrence of either three consecutive repetitions with an incomplete range of motion or the inability to maintain the prescribed tempo. Importantly, participants were blinded to the predefined termination criteria to minimize any psychological influence on performance.

### 2.3. Therapeutic Interventions

Group 1 was treated with TENS using the Multitronic MT-6 (EiE Otwock, Poland) device, with the following parameters: frequency—100 Hz; pulse width—100 μs; rectangle impulse; and amperage up to 30 mA (depending on patient sensory toleration). A single treatment procedure lasted for 30 min. In Group 2, the participants received the sham—TENS. This means that the device generated TENS currents with parameters such as in Group 1 but with intensity values below the threshold of sensation, without causing muscle cramps or tremors. Electrodes were placed on the medial head of the abdominal muscle of the calf along the tibial nerve, in such a way that the flow of the current would cover the place where myotonometric measurements were carried out. Trained physical therapists with clinical experience in the musculoskeletal field performed the procedures.

### 2.4. Assessment—Myotonometry

The MyotonPRO device (Myoton Ltd., Tallinn, Estonia) employed for assessing muscle biomechanical properties is a digital instrument comprising a main body and a 3 mm diameter depth probe, enabling non-invasive evaluation of tissue characteristics [20]. The device operates based on the Mechanical Dynamic Response method, which involves delivering a controlled mechanical impulse to the tissue and recording its dynamic response via oscillation displacement and acceleration signals. These data are then used to calculate biomechanical parameters including muscle tone (Hz), stiffness (N/m), and logarithmic decrement (D)—with the latter reflecting tissue elasticity, where lower values indicate greater elasticity and viscoelasticity. Additional parameters assessed include the relaxation time of mechanical stress (ms) and the Deborah number, which represents the ratio of relaxation time to deformation time and characterizes tissue creep behavior [21]. During measurement, the probe was positioned perpendicular to the target tissue. The device applied a pre-pressure force of 0.18 N, followed by a calibrated mechanical impulse delivered as five consecutive pulses of 0.4 N each, lasting 15 ms, to deform the tissue. The built-in accelerometer recorded tissue oscillations throughout the procedure. To ensure measurement reliability, the coefficient of variation (CV) was monitored for each trial; tests with CVs exceeding 3% were repeated. Each parameter was measured three times per session, and the mean values were used for analysis. The total measurement duration did not exceed 20 s. All measurements and interventions were performed by a physiotherapist experienced in myotonometry and scientific research protocols. Participants were positioned prone, lying face down with knees extended and feet unsupported, hanging off the edge of the examination couch. The ankle joints were maintained in a neutral position, and the lower limbs were stabilized with straps placed just above the popliteal fossa. Measurements targeted the medial head of the gastrocnemius muscle, at a location four finger-widths distal to the popliteal crease, corresponding to the widest part of the muscle belly, following established protocols [22]. To account for anatomical variability, calf circumference was measured with a tape at the designated site, and measurement points were adjusted accordingly.

### 2.5. Measurement of Pain Intensity

Pain intensity was evaluated using the Numeric Rating Scale (NRS), a widely employed tool for pain assessment. The NRS allows patients to rate their pain severity at a given moment on a scale from 0 to 10, where 0 corresponds to ‘no pain’ and 10 represents ‘the worst pain imaginable’ [23]. Participants were instructed to self-report their pain intensity according to this scale.

### 2.6. Statistical Analysis

Mean and standard deviation were calculated to summarize the central tendency and variability of demographic data. The distribution normality of study variables was assessed using the Shapiro–Wilk test, which indicated non-normal distribution. Therefore, all measured variables are presented as medians with interquartile ranges (Q1–Q3). Changes in the measured variables over time (Days 0–4) were analyzed using Friedman’s ANOVA for repeated measures by ranks. Between-group differences (Group 1 vs. Group 2) were examined with the Mann–Whitney U test. Post hoc pairwise comparisons were conducted with Bonferroni correction to control for multiple testing. Effect sizes were calculated as partial eta squared (ηp^2^) and were interpreted according to established thresholds: no effect (0 ≤ ηp^2^ < 0.05), minimal effect (0.05 ≤ ηp^2^ < 0.26), moderate effect (0.26 ≤ ηp^2^ < 0.64), and strong effect (ηp^2^ ≥ 0.64) [24]. An a priori power analysis was performed using G*Power software (version 3.1.9.7; Heinrich-Heine-Universität Düsseldorf, Germany; http://www.gpower.hhu.de, accessed on 10 February 2025) [25]. With a repeated measures ANOVA design incorporating within–between interactions, an effect size of 0.25, an alpha level of 0.05, a power (1–β) of 0.95, and a correlation among repeated measures of 0.5, the required sample size was 36 participants to achieve 95.17% statistical power. To accommodate potential dropouts and maintain balanced group sizes, the sample size was set at 40 subjects. All statistical analyses were conducted using PQStat version 1.8.6. Statistical significance was defined as *p* < 0.05.

## 3. Results

The average age in Group 1 amounted to 34.35 ± 7.29 years of age; in Group 2, it amounted to 35.05 ± 9.76 years of age. The difference was not statistically significant (*p* = 0.935). The average value of the BMI in Group 1 amounted to 23.64 ± 2.96 kg/m^2^, while in Group 2 its value was 25.05 ± 2.79 kg/m^2^. The difference was not statistically significant (*p* = 0.635).

**Figure 2:**
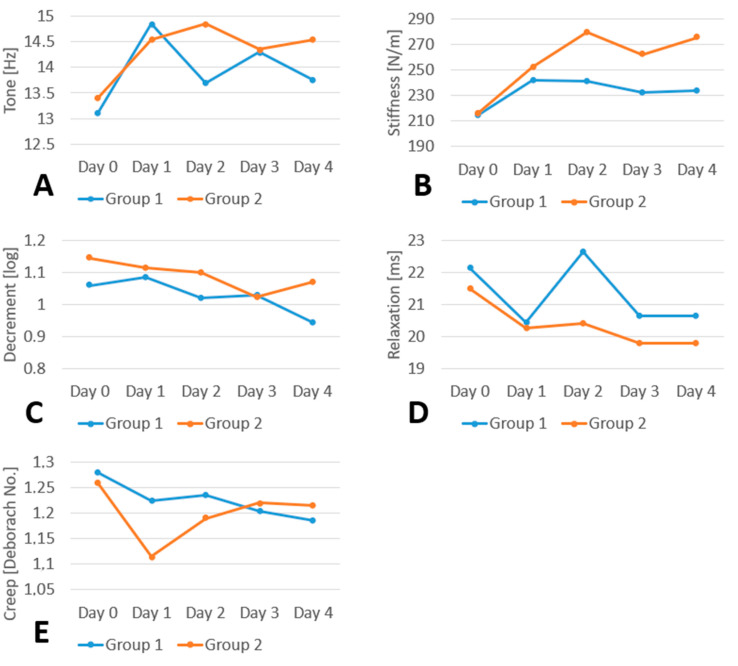
Graphical representation of changes in the biomechanical properties of the gastrocnemius muscle in both study groups from Day 1 to Day 4: (**A**) tone, (**B**) stiffness, (**C**) logarithmic decrement, (**D**) mechanical stress relaxation time, and (**E**) creep (Deborah number).

Within-group comparisons of successive measurements (Days 0–4) were conducted separately for each group. Additionally, between-group comparisons (Group 1 vs. Group 2) were performed for each measurement point. A statistically significant change in muscle tone over time was observed in Group 2 (*p* = 0.001). Post hoc multiple comparisons (Dunn–Bonferroni) that were performed revealed statistically significant differences which were as follows: (Day 0 vs. Day 1), *p* = 0.019; (Day 0 vs. Day 2), *p* = 0.009; (Day 0 vs. Day 3), *p* = 0.019; (Day 0 vs. Day 4), *p* = 0.001. The changes in tone in Group 1 were not statistically significant (*p* = 0.162). Following an initial increase in muscle tone observed in both groups, a reduction in tone values was noted earlier in Group 1—beginning on Day 1—accompanied by overall lower values compared to Group 2. However, by the end of the observation period, tone values in neither group returned to baseline levels. No statistically significant differences in tone values were found between Group 1 and Group 2 at any of the measurement points (Days 0–4) (Table 1A, Figure 2A).

Statistically significant changes in muscle stiffness were observed in Group 2 (*p* < 0.001). Post hoc multiple comparisons using the Dunn–Bonferroni test indicated significant differences between the following time points: Day 0 vs. Day 2 (*p* = 0.001), Day 0 vs. Day 3 (*p* = 0.013), and Day 0 vs. Day 4 (*p* < 0.001). Changes in stiffness in Group 1 turned out not to be statistically significant (*p* = 0.212). After the initial increase in stiffness, which occurred in both groups, stiffness began to decrease earlier, namely from Day 1 in Group 1, and the observed values were lower. A statistically significantly lower stiffness was noted in Group 1 (*p* = 0.008) on Day 4, which was closer to the baseline value, but it never decreased to such a level (Table 1B and Figure 2B).

Decrement values decreased statistically significantly, both in Group 1 (*p* = 0.015) and in Group 2 (*p* = 0.014). Post hoc multiple comparisons (Dunn–Bonferroni) revealed statistically significant differences for the following comparisons: (Day 0 vs. Day 3), *p* = 0.022, and (Day 0 vs. Day 4), *p* = 0.031, in Group 1; and (Day 1 vs. Day 4), *p* = 0.008, in Group 2. There were no statistically significant differences in decrement between Group 1 and 2 on any of the days during which the study was conducted (Day 0–4) (Table 1C and Figure 2C).

The values of relaxation and creep decreased statistically insignificantly in Group 1 (*p* = 0.086 and *p* = 0.185, respectively) and in Group 2 (*p* = 0.105 and *p* = 0.374, respectively). Also, no statistically significant differences were noted between the groups (Table 1D,E and Figure 2D,E).

**Figure 3:**
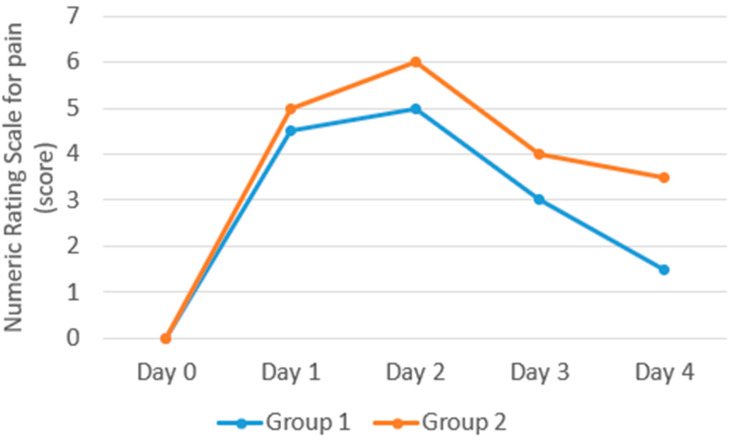
Graphical depiction of pain intensity variations from Day 1 to Day 4 in the respective study groups.

Statistically significant changes in pain intensity were observed in Group 1 (*p* < 0.001) and Group 2 (*p* < 0.001). Post hoc multiple comparisons (Dunn–Bonferroni) revealed statistically significant intensification of pain for (Day 0 vs. Day 2), both in Group 1 (*p* < 0.001) and in Group 2 (*p* < 0.001). From Day 2 on, pain turned out to decrease statistically significantly only in Group 1 in the case of (Day 2 vs. Day 4; *p* < 0.001). At the end of observation (Day 4), a statistically significant (*p* = 0.006) decrease in pain intensity was observed in Group 1 (Table 2 and Figure 3).

## 4. Discussion

The present study evaluated the effects of TENS on the restoration of biomechanical and viscoelastic muscle properties altered during DOMS. These outcomes were compared with a control group receiving a sham treatment. The primary findings are summarized as follows: Treatment procedures employing TENS did not significantly reduce the duration of the increased muscle tone observed in the course of DOMS. This parameter started to decrease, that is, to normalize, one day earlier, in comparison with the control group. In the TENS group, a decrease in stiffness was observed earlier; however, the differences between groups on Days 1, 2, and 3 were not statistically significant, and only on Day 4 were stiffness values significantly lower than in the control group. During the entire test (Days 0–4), the tone and stiffness did not decrease enough to reach the initial values. Elasticity increased significantly both after treatment procedures involving TENS and in the control group, as evidenced by lower decrement values. Logarithmic decrement characterizes the dampening of tissue oscillation. The faster the tissue oscillation fades, the higher the dissipation of mechanical energy induced by the measurement impulse. According to the criteria for the interpretation of parameters, decrement inversely describes elasticity. The values of the parameters that characterize viscoelastic properties, such as relaxation and creep, decreased insignificantly. They changed inversely to tone and stiffness, which was also consistent with the criteria of interpretation: the lower the values of relaxation and creep, the higher the values of tone and stiffness. Relaxation is the time it takes for the muscle or tissue to return to its original state after being stretched or deformed. A shorter relaxation time indicates a quicker return to baseline, meaning a more elastic tissue. Greater tissue tension and stiffness result in faster shape recovery, indicating a lower relaxation value. The ratio of relaxation and deformation time characterizes creep. It is the gradual elongation of tissue over time when placed under constant tensile tress. Increased tissue tension and stiffness lead to greater resistance to creep, resulting in a lower creep value. To wrap up, treatment procedures involving TENS appear to have limited efficiency in restoring normal biomechanical and viscoelastic properties in the course of DOMS. However, a significant reduction in pain level was observed when TENS was applied.

There are no studies available in the literature which refer to the effect that TENS may have on biomechanical and viscoelastic parameters of muscles in the course of DOMS. This prevents us from comparing our results with other such results. However, our study is the first one intended to assess the impact of TENS on muscle biomechanics by means of myotonometry. On the other hand, there are numerous studies available which assess the efficiency of analgesic effects of TENS in the course of DOMS.

Craig et al. [26] evaluated the hypoalgesic effects of TENS during the acute phase (72 h) of experimentally induced DOMS in a cohort of 48 participants, who were randomly assigned to one of four groups: control, placebo, low-frequency TENS (200 μs pulse duration; 4 Hz), or high-frequency TENS (200 μs; 110 Hz). DOMS was induced in all subjects through standardized repeated eccentric exercise targeting the non-dominant elbow flexors. The results demonstrated only sporadic and inconsistent effects of high-frequency TENS (110 Hz) on resting joint angle and flexion measures, with no significant changes observed for other assessed variables. Overall, these findings do not provide strong evidence supporting the hypoalgesic efficacy of TENS on DOMS-related pain using the parameters tested. Besides TENS, other forms of electrotherapy have also been used to alleviate DOMS symptoms. Sara et al. [27] demonstrated the higher effectiveness of Micro-Current Electrical Therapy (MET) compared to conventional TENS. In contrast to these findings, a systematic review and meta-analysis conducted by Menezes et al. [13], which included 14 randomized clinical trials (*n* = 435), did not confirm the effectiveness of electrical stimulation in the treatment or prevention of DOMS. The authors pointed to the low quality of evidence and the lack of clear recommendations regarding the use of this method.

These findings indicate that the muscle weakness observed in DOMS is unlikely to be primarily due to pain-induced inhibition. The data further imply that therapeutic modalities effectively alleviate pain and muscle spasms related to DOMS; however, a reduction in pain intensity may not reliably reflect the restoration of muscle strength [28].

Wiecha et al. [29] reviewed the current scientific data concerning clinical outcomes (efficacy, safety) of physiotherapy treatment procedures for DOMS in healthy adults. Available databases were analyzed, MEDLINE, MEDLINE, Embase, Cochrane Database of Systematic Reviews, Epistemonikos and PEDro, comprising the period from 1998 to 2024. The results showed that there was no consensus as to the standard of physiotherapeutic care in the case of DOMS. Also, Menezes et al. [13] in their study emphasize that the effectiveness of electrical stimulation (ES) in prevention or treatment of DOMS and its effect on regeneration is unclear. The authors conducted a review of the databases (PubMed, Medline, CENTRAL, EMBASE, CINAHL, PsycINFO, PEDro, LILACS, SPORTDiscus) for randomized control studies (RCT). The analyzed research results indicate that ES neither prevents nor treats DOMS, shown as not being helpful in muscle recovery. There are no recommendations to support the use of ES in DOMS. This means that electrical stimulation proves to be not beneficial for the analyzed population, according to the treatment protocols used. Therefore, further randomized trials with the same approach are unlikely to give promising results.

Gussoni et al. [30] evaluated the efficacy of electrical stimulation (ES) treatment (without inducing muscle contraction) in 11 healthy volunteers, following eccentric upper limb exercises, as well as in 14 ultra-endurance athletes. There were no significant differences in recovery times between the sham therapy groups and the ES group. The authors concluded that both the efficacy of ES in dealing with DOMS and its effect on muscle recovery remain unclear.

Other forms of physiotherapy are also used to alleviate symptoms associated with DOMS. The application of paired-associative electromagnetic stimulation in athletes with DOMS following eccentric exercise resulted in improved physical performance parameters of the lower limbs, such as strength and running speed, as well as a reduction in pain intensity [31]. In another study, Balasubramaniyam et al. [32] observed a significant reduction in pain following cryotherapy combined with TENS compared to cryotherapy alone. A systematic review and meta-analysis conducted by Nahon et al. [4] which included 121 studies, showed that methods such as cryotherapy, phototherapy, vibration, ultrasound, massage, and active exercise have beneficial effects in the management of DOMS-related pain. Despite the positive results, the quality of evidence remains low, and the high level of heterogeneity in research protocols makes it difficult to clearly assess the effectiveness of the interventions [33]. In the prevention and mitigation of delayed onset muscle soreness (DOMS), dietary supplements are also employed, although with varying degrees of efficacy [34]. Non-steroidal anti-inflammatory drugs (NSAIDs) are frequently consumed by athletes to manage DOMS, although their effects on muscle soreness and performance remain unclear [35].

On the basis of our own research and observations made by other researchers, it appears that electric nerve stimulation treatment (TENS) may affect the functioning of the nervous system and muscles, but its impact on the biomechanical parameters of muscles seems to be limited [4,10]. In most cases, such electric nerve stimulation reduces pain, which may indirectly promote the improvement of muscle biomechanics. However, more comprehensive rehabilitation is necessary for the complete normalization of biomechanical and viscoelastic parameters of affected muscles in DOMS. TENS treatment helps relieve the pain associated with DOMS, though, allowing for faster recovery [11,28].

In the sham group, participants were exposed to sub-threshold electrical stimulation—a current that was not detectable to them. This approach is widely used and is effective in maintaining blinding. However, it is important to recognize that current research indicates that even imperceptible forms of electrical stimulation, such as TENS and transcranial electrical stimulation (TES), can induce subtle yet meaningful physiological effects. These techniques exert their influence by altering neuronal firing patterns, engaging various neurotransmitter systems, and modulating pain pathways. Given this, such stimulation may not be entirely biologically inert [36,37].

It seems justified to conclude that future research should also consider assessing other types of electric stimulation or use of currents, such as interference currents, Kotz currents, or neuromuscular electrostimulation (NMES). The latter NMES also uses an impulse current, but unlike TENS, its task is to stimulate motor and sensory fibers to activate muscles. Neuromuscular electrostimulation uses electrical impulses to induce muscle contraction, so that muscles can operate without involving the patient’s strength. Simultaneous work of muscles and their electrical stimulation allow for obtaining therapeutic effects faster. Circulation improves in such cases, thanks to which better muscle nutrition is provided, which translates into faster post-DOMS recovery [38].

### Study Limitations

This study has several notable limitations that should be taken into consideration when interpreting the results. Most importantly, the relatively small sample size limits the generalizability of the findings and reduces the statistical power of the analyses. As such, the results should be interpreted with caution, and further studies with larger cohorts are recommended to confirm these preliminary findings. Additionally, the baseline values of the measured parameters were assumed to be normative and were self-monitored. The lack of objective assessments of body composition—particularly in terms of adiposity content—represents a significant methodological limitation. The thickness of the subcutaneous fat layer was not measured, which could have affected the efficacy and depth of TENS signal penetration and influenced the obtained myotonometry measurement values [39,40].

There was also a potential risk of unmeasured and accidental unblinding, as participants might have perceived qualitative differences between the active and placebo TENS. Importantly, the effectiveness of the blinding procedure was not assessed by directly asking participants whether they believed they had received active treatment, which limits the ability to rule out expectancy effects. Moreover, pain intensity was measured solely using the self-reported NRS, which, although widely used, is inherently subjective. The absence of complementary objective or behavioral measures of pain response may have influenced the accuracy and reliability of the assessment of TENS analgesic efficacy.

Given these limitations, the authors suggest that future research should aim to replicate the study using a larger and more diverse sample, and with enhanced methodological rigor. In particular, it would be valuable to conduct a similar experiment designed to evaluate the prophylactic potential of TENS in preventing the development of DOMS symptoms, with the intervention applied immediately after exercise on the same day. Such a design could provide further insight into the optimal timing and efficacy of TENS application for post-exercise muscle soreness.

## 5. Conclusions

Current treatment procedures using TENS have limited effectiveness in restoring the baseline biomechanical and viscoelastic muscle parameters that were altered in DOMS. TENS turns out not to be significantly more effective in normalizing tone, stiffness, elasticity, and relaxation, as well as creep, than a placebo is, applied in the form of a sham therapy. Stimulation by means of TENS significantly relieves pain symptoms experienced in DOMS.

## Figures and Tables

**Table 1:** Changes in gastrocnemius muscle biomechanical properties measured using the MyotonPRO device across specific time points (Days 0–4) in both study groups: (A) tone, (B) stiffness, (C) logarithmic decrement, (D) relaxation time, and (E) creep (Deborah number).

	Day 0	Day 1	Day 2	Day 3	Day 4		
	Median (Q1–Q3)					(ηp^2^)	** *p*
(A) Tone [Hz]							
**Group 1**	13.1 (12.18–14.38)	14.85 (13.75–17.23)	13.7 (13.4–15.63)	14.3 (13.3–15.53)	13.75 (13.08–14.83)	0.08	0.162
**Group 2**	13.4 (12.7–13.93)	14.55 (13.28–16.23)	14.85 (13.76–16.13)	14.35 (13.25–16.25)	14.55 (13.8–16.55)	0.23	**0.001**
**(ηp^2^)**	0.08	0.09	0.18	0.05	0.28		
* *p*	0.578	0.533	0.261	0.755	0.078		
**(B) Stiffness [N/m]**							
**Group 1**	215 (187.25–240.5)	242 (221–258.25)	241 (229.5–265.5)	232.5 (202.25–277.75)	234 (194–259.75)	0.07	0.212
**Group 2**	216 (193.75–266.25)	252.5 (202–315)	279.5 (235–312)	262 (232.5–308.25)	275.5 (237.5–308.25)	0.27	**<0.001**
**(ηp^2^)**	0.08	0.07	0.23	0.22	0.42		
* *p*	0.635	0.674	0.140	0.171	**0.008**		
**(C) Decrement [log]**							
**Group 1**	1.06 (1–1.15)	1.09 (0.93–1.2)	1.02 (0.92–1.25)	1.03 (0.96–1.27)	0.95 (0.86–1)	0.16	**0.015**
**Group 2**	1.15 (1.01–1.37)	1.12 (1.01–1.24)	1.1 (0.98–1.22)	1.03 (0.93–1.19)	1.07 (0.91–1.21)	0.16	**0.014**
**(ηp^2^)**	0.18	0.17	0.07	0.09	0.3		
* *p*	0.249	0.273	0.664	0.578	0.058		
**(D) Relaxation [ms]**							
**Group 1**	22.15 (20.43–25.48)	20.45 (19.18–23.43)	22.65 (19.03–23.63)	20.65 (19.2–23.13)	20.65 (18.75–22.7)	0.1	0.086
**Group 2**	21.5 (19.55–22.75)	20.25 (16.1–22.2)	20.4 (17.75–22)	19.8 (16.4–21.95)	19.8 (15.88–22.23)	0.1	0.105
**(ηp^2^)**	0.19	0.13	0.29	0.13	0.13		
* *p*	0.239	0.393	0.067	0.424	0.394		
**(E) Creep [Deborach No.]**							
**Group 1**	1.28 (1.15–1.47)	1.23 (1.14–1.39)	1.24 (1.1–1.37)	1.21 (1.13–1.28)	1.19 (1.07–1.26)	0.08	0.185
**Group 2**	1.26 (1.11–1.4)	1.12 (0.99–1.39)	1.19 (1.06–1.29)	1.22 (1.09–1.33)	1.22 (1.08–1.29)	0.05	0.374
**(ηp^2^)**	0.09	0.16	0.12	0.09	0.11		
* *p*	0.551	0.303	0.432	0.569	0.498		

* *p*: Mann–Whitney U Test; ** *p*: Friedman’s ANOVA.

**Table 2:** Evaluation of pain levels at designated time points in each of the study groups.

	Day 0	Day 1	Day 2	Day 3	Day 4		
	Median (Q1–Q3)					(ηp^2^)	** *p*
Numeric Rating Scale (NRS) for Pain							
**Group 1**	0 (0–0)	4.5 (2.75–6)	5 (3.75–7)	3 (1.75–4)	1.5 (1–2)	0.76	**<0.001**
**Group 2**	0 (0–0)	5 (3–7)	6 (4.5–7.5)	4 (2.5–7)	3.5 (1.75–5)	0.55	**<0.001**
**(ηp^2^)**	NA	0.07	0.17	0.34	0.56		
* *p*	NA	0.681	0.427	0.328	**0.006**		

* *p*: Mann–Whitney U Test; ** *p*: Friedman’s ANOVA; NA—not applicable.

## Data Availability

Data are available upon reasonable request to the corresponding author.
